# Variation on the Microstructure and Mechanical Properties of Ti-Al-N Films Induced by RF-ICP Ion Source Enhanced Reactive Nitrogen Plasma Atmosphere

**DOI:** 10.1186/s11671-020-03354-5

**Published:** 2020-05-24

**Authors:** Dongke Li, Lixia Xia, Lian Yan, Yunqing Cao, Zhangyin Zhai, Guibin Chen

**Affiliations:** 1 Physics Department, Huaiyin Normal University, Huai’an, 223300 China; 2 School of Electronic Science and Engineering, National Laboratory of Solid State Microstructures, Collaborative Innovation Center of Advanced Microstructures, Jiangsu Provincial Key Laboratory of Advanced Photonic and Electronic Materials, Nanjing University, Nanjing, 210000 China; 3 Huaiyin Normal University, Huai’an, 223300 China; 4 School of Physical Science and Technology, Yangzhou University, Yangzhou, 225000 China

**Keywords:** Ti-Al-N, Nitrogen plasma density, RF-ICPIS, microstructure

## Abstract

Acquiring the optimum growth conditions of Ti-Al-N films, the effects of gas atmosphere, especially the reactive plasma on the material microstructures, and mechanical properties are still a fundamental and important issue. In this study, Ti-Al-N films are reactively deposited by radio frequency inductively coupled plasma ion source (RF-ICPIS) enhanced sputtering system. Different nitrogen gas flow rates in letting into the ion source are adopted to obtain nitrogen plasma densities and alter deposition atmosphere. It is found the nitrogen element contents in the films are quite influenced by the nitrogen plasma density, and the maximum value can reach as high as 67.8% at high gas flow circumstance. XRD spectra and FESEM images indicate that low plasma density is benefit for the film crystallization and dense microstructure. Moreover, the mechanical properties like hardness and tribological performance are mutually enhanced by adjusting the nitrogen atmosphere.

## Introduction

Due to the respectable mechanical properties like high hardness, anti-corrosion, and superior oxidation resistance, Ti-Ai-N has attracted much attraction in dry and high speed cutting as the surface protection films for machine tools [1–3]. Nowadays, varieties of techniques have been developed for the fabrication of Ti-Al-N films including chemical vapor deposition [4], arc evaporation [5], ion plating [6], and reactive DC/RF sputtering [7]. During Ti-Al-N film deposition progress, the gas atmosphere is quite important and complex [8–12]. For example, in the reactive sputtering deposition, the non-equilibrium nitrogen plasma is not only depended on the argon or nitrogen ions concentration, but also affected by the secondary electrons density or total gas pressure that aggravate the difficulties to understand the reactive nitrogen atmosphere induced the variation on the Ti-Al-N film properties. Jeong et al. have reported the growth morphology of Ti-Al-N films that was influenced by nitrogen flow rates [13]. Irudayaraj et al. have found the deposition rate, grain size, and the ratio of concentration of Ti to Al of the deposited Ti-Al-N films decreased with increasing N_2_ flow rate [14]. Because of the importance to acquire valuable knowledge for the selection of optimum growth conditions, the effects of nitrogen atmosphere, especially the reactive plasma density on the material element contents and corresponding microstructures and mechanical properties of Ti-Al-N, are still need further exploring.

In our previous work, radio frequency inductively coupled plasma ion source (RF-ICPIS) has been verified to enhance the Ti-Al-N deposition by dropping the argon gas ionization temperature and increasing the ionization rates [15]. In this paper, we straightly ionize nitrogen gas in a discharged RF-ICPIS cavity, and a dense nitrogen plasma beam is directly introduced into the reactive chamber to take part in reactive deposition. Comparing with traditionally RF/DC sputtering system, the nitrogen plasma density provided by RF-ICPIS can be readily controlled by changing the RF powers or gas flow rates. The influences of nitrogen plasma variation on the element contents, microstructures, surface morphologies, as well as the mechanical properties of Ti-Al-N films, are studied and discussed.

## Method

### Film Deposition

Ti-Al-N films were deposited on Si (100) and mirror-polished stainless steel by RF-ICP ion source enhanced magnetron sputtering system with the growth temperature at 200 °C. Substrates were placed on a rotating specimen holder (20 rpm) which was perpendicular to a Ti_0.5_Al_0.5_ compound target with purity of 99.9%. After pumping sputtering chamber to a base pressure lower than 1.0 × 10^−4^ Pa and target surface cleaning by argon ions, the buffer layer of Ti-Al was then deposited on substrates by DC sputtering. Following, the nitrogen plasma produced from RF-ICPIS was introduced nearby the substrates to attend the reactive deposition for Ti-Al-N film. The RF-ICPIS power was controlled at 50 W, and nitrogen gas flow rates in letting into the ion source were varied from 5 to 25 sccm to alter nitrogen plasma densities and obtain different sputtering and deposition atmosphere. Meanwhile, the DC sputtering current was fixed at 0.4 Å, and the total gas pressure of sputtering chamber was kept at 0.5 Pa. Detailed growth parameters are listed in Table 1.

**Table 1:** Growth parameters of Ti-Al-N films during deposition progress

Samples	N_2_ flows (sccm)	RF-ICPIS power (W)	Ar flows (sccm)	DC current (A)	Total gas pressure (Pa)	Deposition temperature (^o^C)	Film thickness (μm)
S1	5	50	15	0.4	0.5	200	0.86
S2	10	50	15	0.4	0.5	200	0.94
S3	15	50	15	0.4	0.5	200	1.00
S4	20	50	15	0.4	0.5	200	1.33
S5	25	50	15	0.4	0.5	200	1.56

### Characterization

Elements contents were characterized by energy dispersive spectrometer (EDS, Oxford X-Max 50), and crystal structures of Ti-Al-N films were revealed by x-ray diffraction (XRD, Bruker D8 Advance) with a Cu Kα radiation (*λ* = 1.54056 Å). Surface morphologies and cross-sectional microstructure of films were measured by field emission scanning electron microscopy (FESEM, ZEISS Ultra 55). Atomic force microscopy (AFM, Asylum Research) was applied to measure the surface root-mean-square (RMS). Pyramidal diamond tip was adopted for the nano-indentation test, and the hardness was measured by MTS Nano Indenter XP and calculated through the Oliver-Pharr indentation method. Each samples, ten separated point measurements, were taken to get a mean hardness value. A conventional ball-on-disc wear apparatus with a sliding speed of 0.2 m/s under a load of 10 N was used to measure the friction coefficient.

## Results and Discussion

**Fig. 1:**
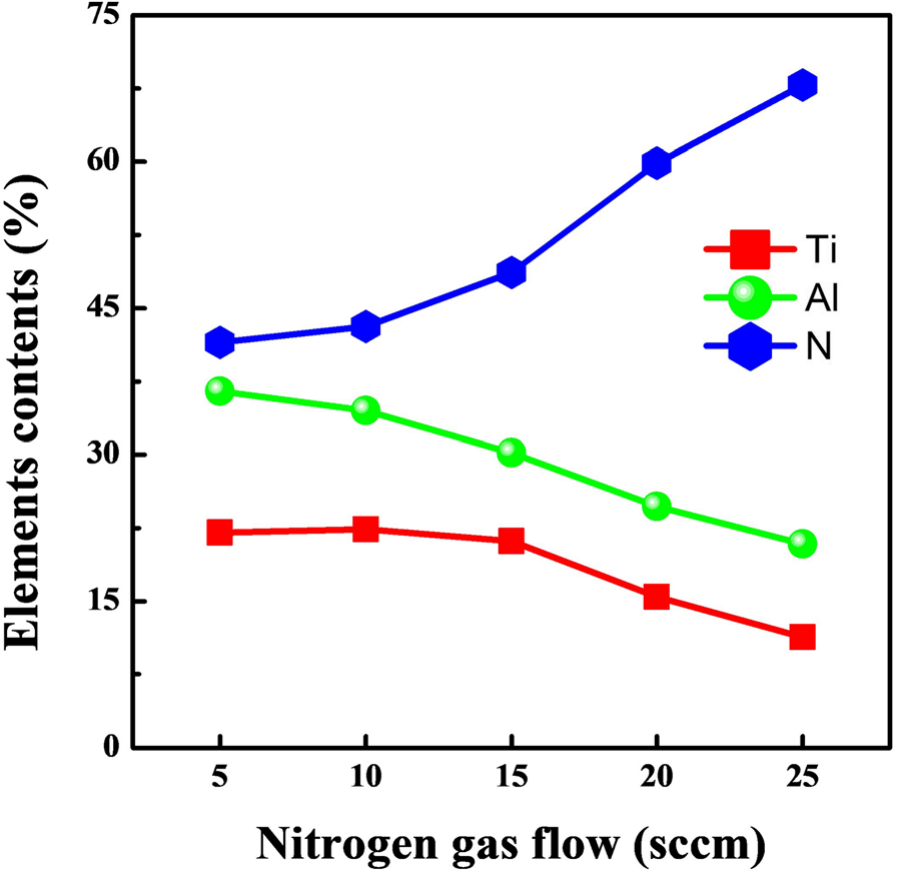
Element contents of Ti-Al-N films fabricated at various nitrogen gas flow rates

Figure 1 shows the Ti, Al, and N element contents in Ti-Al-N films deposited at different nitrogen gas flow rates. The N contents in Ti-Al-N films are monotonically improved with the increase of nitrogen gas flows admitted into the ion source. Within the low nitrogen gas flow region (5–15 sccm), N contents are kept in 45–50%, and the (Ti + Al)/N rations are similar to the (Ti, Al) N constructure. When further improve the nitrogen gas flow rates from 15 to 25 sccm, the N contents in Ti-Al-N films are rapidly increased. The maximum value of 67.8% is obtained at 25 sccm, which is beyond 50% in the common (Ti, Al) N or Ti_x_Al_1-x_N microstructure, which indicate phase transitions are occured in S4 and S5. Ti and Al contents in Ti-Al-N films both exhibit opposite trend to N element as function of nitrogen gas flow rates. For all the films, Al contents are higher than Ti, which is similar with the results of Ti-Al-N films deposited under enhanced plasma atmosphere. The content difference between Al and Ti elements can be attributed to the difference of sputtering yield between Al atom with a light quality and Ti atom. Additionally, Al atoms are easily oozing to the films surface regions, which also could led the detected Al contents are slightly higher than the actual values in the films inner [16].

**Fig. 2:**
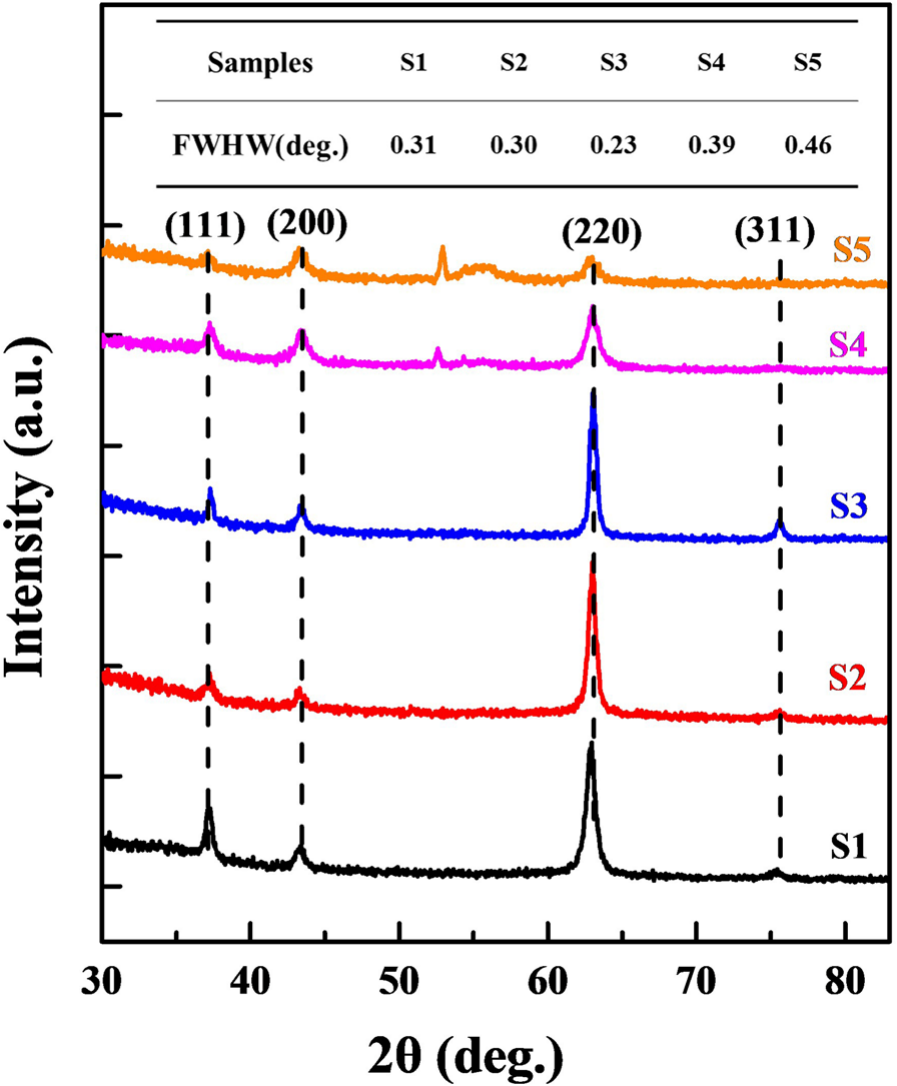
XRD spectra of S1–S5 samples. Insert table is the FWHW value of (220) for S1–S5

XRD is performed for the samples deposited at Si (100) wafers. As shown in Fig. 2, S1–S5 exhibit the typical NaCl type face-centered cubic (f.c.c) structure with multiple diffraction plans orientated at (111), (200), (220), and (311), excepting for (311) for S4 and S5 samples deposited at high gas flow rates [17]. For f.c.c Ti-Al-N structure, (111) is the densely packed plane with a lowest surface energy, while the (200) and (220) followed. In S1–S5, all deposited Ti-Al-N exhibit the (220) preferred orientation rather than (111). In our previous study, we found the film deposition rates were improved in RF-ICPIS enhanced sputtering system. This lead to the decreased migration time of adatoms on substrates and favor the growth of (220) crystals planes with higher surface energy, at the expense of others because of its higher ledge density and then shorter diffusion distance to the relative lower energy sites [13]. Additionally, lattice distortion induced by Al atoms incorporating with a high concentration also contribute to the (220) preferential growth rather than (111) [18]. The intensities and FWHM of (220) peaks also reveal the dependence of crystallization on the nitrogen gas flow rates. In the low gas flow range (5–15 sccm), the intensity and FWHM are improved when increase the nitrogen flow rate, which indicate the Ti-Al-N crystalline grain sizes in films that are decreased and films crystallization qualities that are enhanced [19]. For S4 and S5, the reduced peak intensities and enlarged FWHM that may verified high gas flow rates (20–25 sccm) are adverse to the Ti-Al-N films qualities.

Degenerated Ti-Al-N crystal quality at high nitrogen gas flow rates is the result of nitrogen plasma variation originating from RF-ICPIS. More nitrogen atoms can be ionized when increase the nitrogen flow rates into the ion source, and the corresponding plasma densities in sputtering chamber are also enhanced. The electron concentrations near the substrates are measured by Langmuir probe. Calculated results show that the mean electron concentrations vary from 1.5 × 10^16^ to 2.7 × 10^16^ cm^−3^ with the nitrogen gas flow rates increasing from 5 to 25 sccm. The improved electron concentrations indicate the plasma densities are improved during deposition progress. Additionally, the voltages of the sputtering source with a fixed sputtering current of 0.4 A during deposition are 482, 461, 443, 408, and 376 V for 5, 10, 15, 20, and 25 sccm respectively. The diminution of the impedance between substrates and target also reflect the enhanced plasma atmosphere. High plasma density improves the collisions between atoms that led the mean free path of sputtered metal atoms and film deposition rates decrease. Followed, the adsorbed atoms on the substrates have more time to migrate and nucleate and contribute to the film crystallizations. When nitrogen flow rates exceed a certain threshold, although the plasma densities are further improved, more nitrogen atoms would not be fully ionized under a fixed ion source power of 50 W [20]. Comparing with fully ionized nitrogen atoms, those un-fully ionized atoms are more close to substrate and would directly take part in films nucleation growth. As a result, the nitrogen contents in Ti-Al-N films are quite beyond the stoichiometric ratio.

**Fig. 3:**
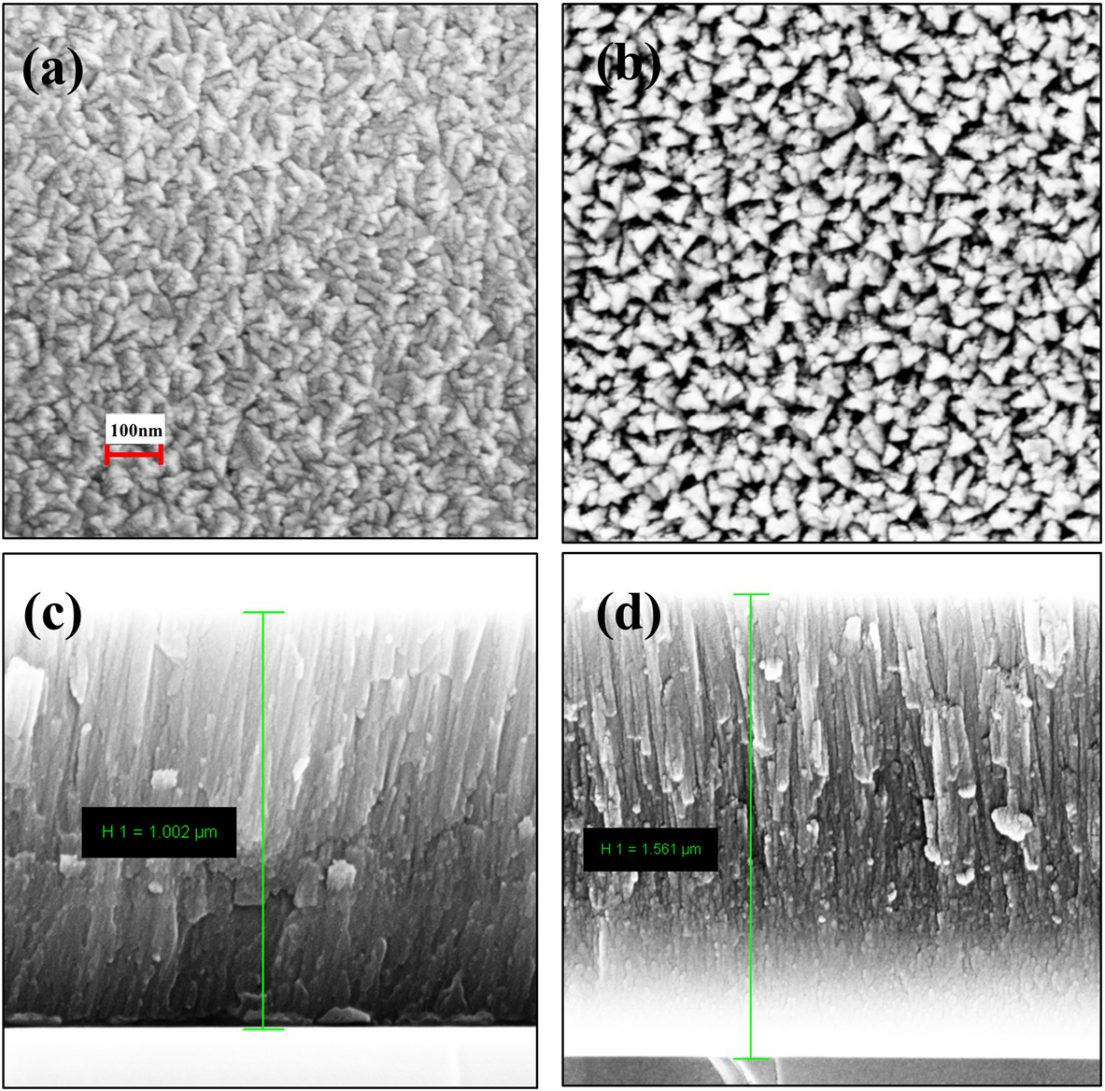
Planar and cross-sectional FESEM images of S3 (**a**, **c**; 15 sccm) and S5 (**b**, **d**; 25 sccm)

Figure 3 shows the planar and cross-sectional micro-morphologies of Ti-Al-N films observed by FESEM. The Ti–Al–N surface morphologies exhibit as the typical tripartite cone grains [21]. Comparing with Fig. 3a, b, we can find the film deposited at low nitrogen plasma density (S3) has smaller grain sizes and denser surfaces than S5 deposited at high plasma density, which is also in accord with the XRD results. In low plasma density atmosphere, fully ionized nitrogen atoms and sufficient migration time of adatoms promote the growth and crystallization of Ti-Al-N and contribute to the denser surface. The microstructures of comparative sample CS3, which is deposited through the traditional method of ionizing argon gas and has the same experimental parameter with S3, are also studied (see supplementary material). Comparing with S3, CS3 shows a looser and rougher planar surface, and many voids appear among the grains boundaries. Meanwhile, the CS3 has the smaller film thickness than S3. The reasons are mainly attributed to the differences of deposition atmosphere between those two gas ionization methods. By straightly ionizing nitrogen gas in RF-ICPIS, the gas ionization temperature can be efficiently dropped and high-density nitrogen plasma can be obtained. Consequently, the adatoms on substrates would have high migration energy and be benefit for the growth and crystallization of Ti-Al-N films. In the cross-sectional FESEM images, columnar structures are clearly observed for Ti-Al-N films deposited at 15 and 25 sccm, and the films reveal the thickness of 1.002 and 1.561 μm, respectively. In high plasma density circumstance, the film deposition rate is increased by 50%. The results are different from the enhanced atoms scattering induced low deposition rate and mainly arise from the rapid growth of weakly bonded nitride associated with those un-fully ionized nitrogen atoms. Meanwhile, the columnar structures of S3 exhibit grained and denser nanostructures, and the S5 sample reveals the columnar structures with voids and boundaries throughout the film. It is further evident that high nitrogen plasma density is adverse to the Ti-Al-N films crystallization qualities.

**Fig. 4:**
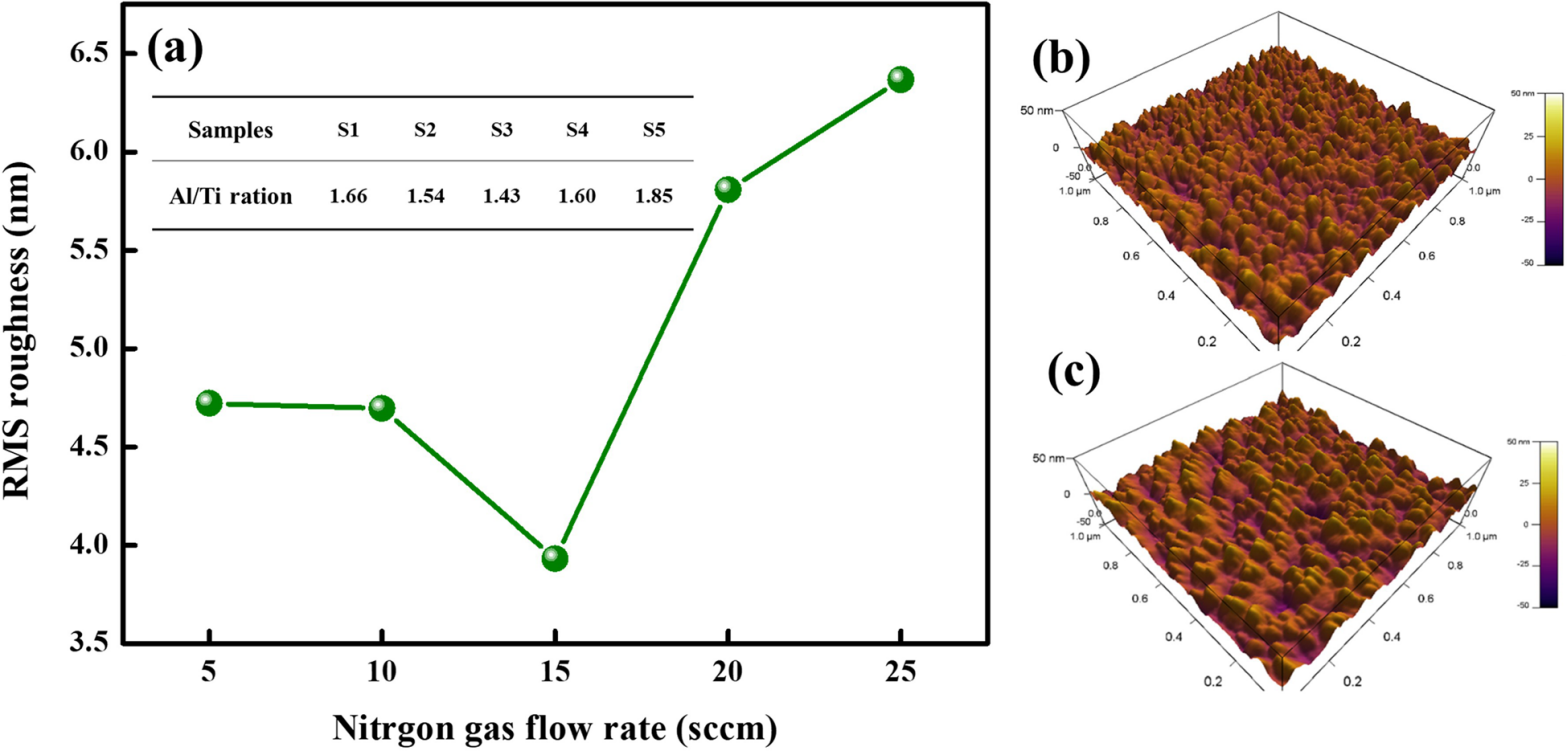
**a** RMS roughness values of Ti–Al–N films as a function of nitrogen gas flow rates. Insert table is the values of Al/Ti rations; **b** and **c** are the AFM images of S3 and S5 samples, respectively

Further, the roughness of Ti-Al-N films deposited at various nitrogen gas flow rates is studied by AFM, and the root-mean-square (RMS) roughness values are plotted in Fig. 4. The roughness is firstly reduced and then increased with the increase of nitrogen gas flow rates, and the minimum value of 3.932 nm is obtained at 15 sccm. Meanwhile, as shown in Fig. 4c, we can find the film surface of S5 sample that is filled with swollen particles and the ravine between particles that are quite deep. The rougher surfaces of S5 can be attributed to the poor crystal qualities and sparse surfaces. In Ti–Al–N crystal structure, the Al/Ti ratio is also an important factor that Al atoms occupy lattice sites of Ti atoms and introduces lattice defects to influence the microstructure and mechanical properties [18, 22]. Calculated Al/Ti ratios based on EDS tests are 1.66, 1.54, 1.43, 1.60, and 1.85 for S1–S5, respectively. The excellent crystal quality and low Al/Ti ratio are contributed to the smoothest surface of Ti-Al-N sample deposited at 1 sccm. High Al/Ti ratios aggravate lattice distortion structure defects in the S5 films and exacerbate the surface roughness.

**Fig. 5:**
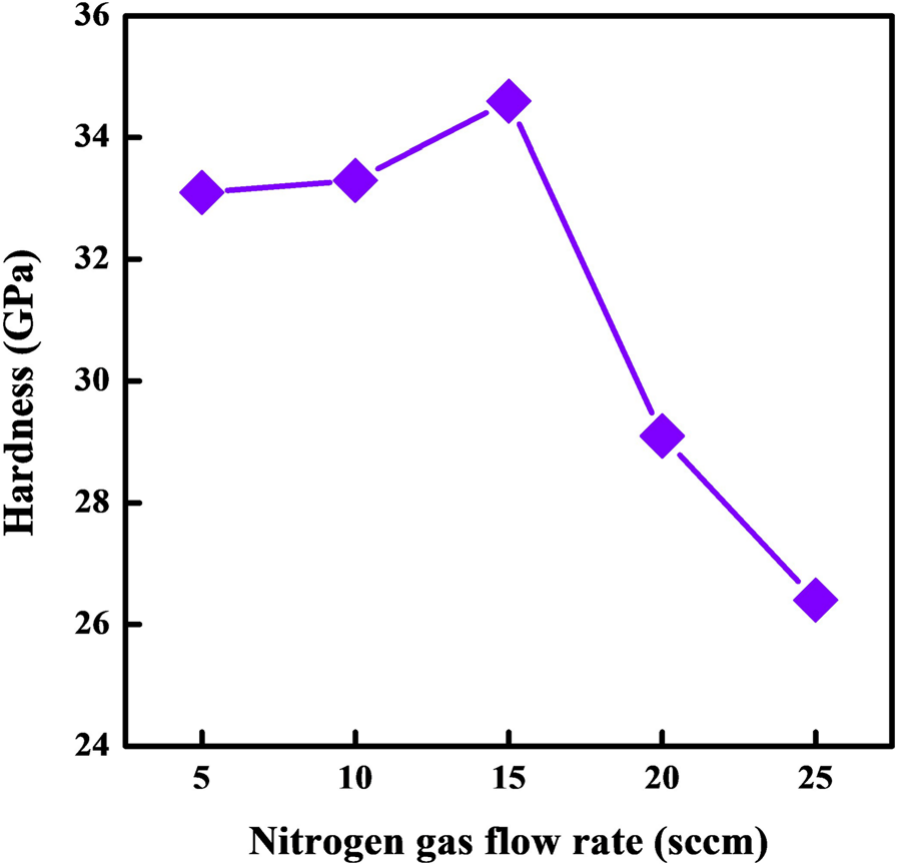
Hardness of Ti-Al-N films deposited at different nitrogen gas flow rates

The hardness of Ti-Al-N films deposited on stainless steel substrates under different nitrogen gas flow rates are shown in Fig. 5. The hardness of Ti-Al-N films obtained at 5, 10, 15, 20, and 25 sccm are 33.1, 33.3, 34.6, 29.1, and 26.4 GPa, respectively. In the low nitrogen flow range, the hardness of Ti-Al-N films is quite higher than the traditional Ti-N material. The improved hardness of Ti-Al-N is mainly originated from the microstructure evolution through the introduction of Al contents that Al atoms occupying part lattice sites of Ti atoms cause lattice defeat and increase internal stress of films. Additionally, the Al/Ti ratios of S1–S5 samples are higher than the Ti-Al-N materials fabricated at conventional sputtering system since the RF-ICPIS technique can drop the gas ionization temperature and increase the ionization rate during the reaction sputtering, also convert the sputtering yield of metal particles [23]. High Al/Ti ratios that induced lattice distortion also cause the dislocation motion resistance and difficult slip movement, which can jointly contribute to the excellent hardness performance of Ti-Al-N films deposited at low nitrogen flow rates. Meanwhile, the optimized crystallization and decreased grain sizes further promote the hardness to the maximum of 34.6 GPa at 15 sccm.

**Fig. 6:**
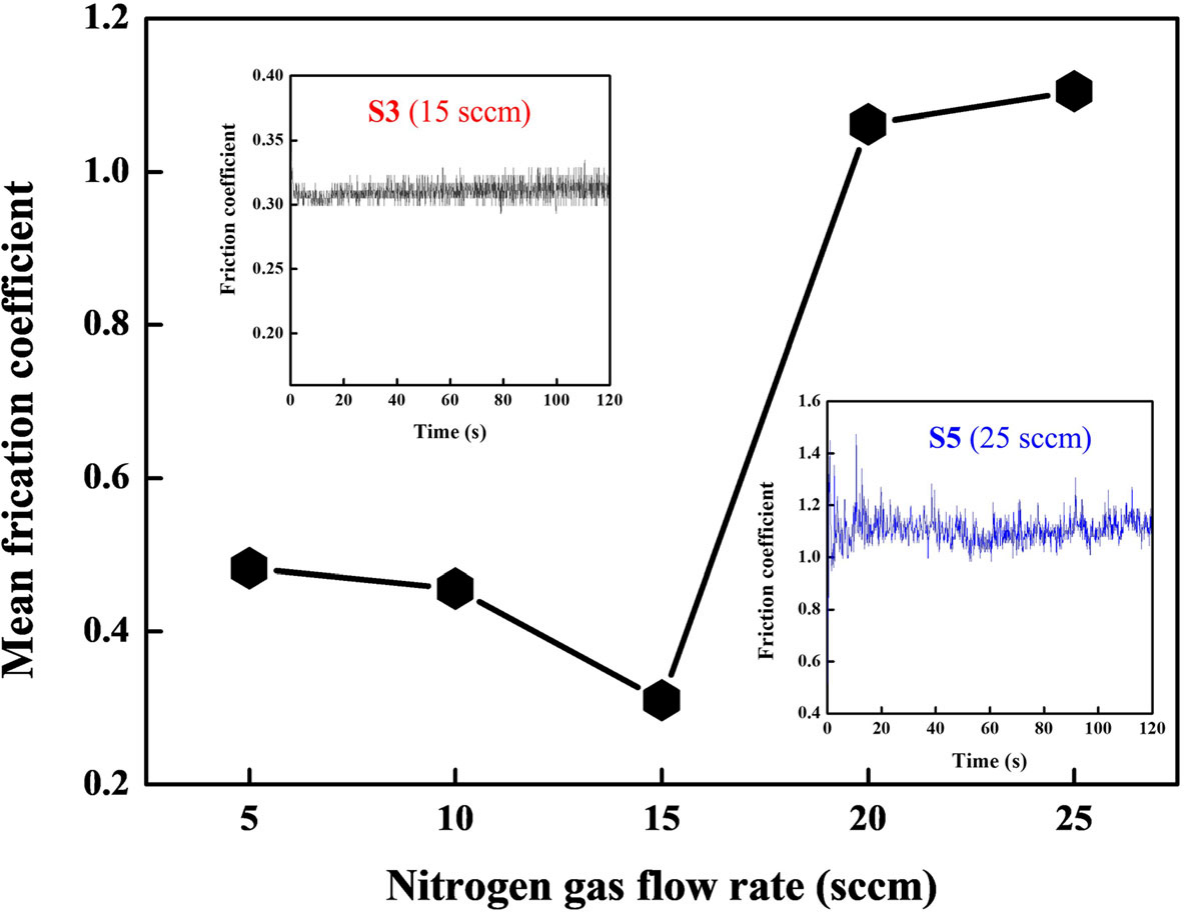
Mean friction coefficient of Ti-Al-N films deposited at different nitrogen gas flow rates. Inserts are the friction coefficient curve of S3 and S5, respectively

Friction properties of Ti-Al-N films are also studied by the ball-on-disc wear apparatus, and the mean friction coefficient of Ti-Al-N films is plotted in Fig. 6. The variation of friction coefficients depending on nitrogen gas flow rates is similar with the RMS roughness. Obviously, the smooth surfaces and dense cross-sectional nanostructures of Ti-Al-N films deposited at low nitrogen gas flow rates are benefit for the surface tribological performance. Meanwhile, the S1–S3 show smaller mean friction coefficient than CS3 (see supplementary material).

## Conclusion

In summary, the microstructure and mechanical properties of Ti-Al-N films deposited at different nitrogen plasma atmosphere are studied. It is found that the element contents in the Ti-Al-N films are quite influenced by the nitrogen plasma density. Low plasma density is benefit for the film crystallization qualities and microstructure, which is supported by XRD spectra and FESEM. Additionally, the surface roughness and mechanical properties like hardness and friction coefficient can be further optimized at appropriate plasma density range. At high plasma density, the excessive nitrogen contents in the films can induce metastable nitride phase, and be responsible for the loose microstructure and aggravated mechanical performance. Our results would be an efficient way to further understand the deposition atmosphere-related growth mechanism of Ti-Al-N films.

## Supplementary information

**Additional file 1: Fig. S1.** (a) Planar, (b)cross-sectional FESEM images and (c) friction coefficient curve of CS3, which are deposited through the traditional method of ionizing argon gas and has the same experimental parameter with S3.

## Data Availability

All data are fully available without restriction.

## Abbreviations

RF-ICPIS: Radio frequency inductively coupled plasma ion source
DC: Direct current
EDS: Energy dispersive spectrometer
XRD: X-ray diffraction
FESEM: Field emission scanning electron microscopy
AFM: Atomic force microscopy
FWHM: Full width at half maximum
RMS: Root-mean-square
