# Supplementary material for: Variation on the Microstructure and Mechanical Properties of Ti-Al-N Films Induced by RF-ICP Ion Source Enhanced Reactive Nitrogen Plasma Atmosphere
Source: Nanoscale Res Lett. 2020 May 24;15:119. doi: 10.1186/s11671-020-03354-5 (PMC7246271; doi:10.1186/s11671-020-03354-5)
Supplement: Supplementary file 1 — Additional file 1: Fig. S1. (a) Planar, (b)cross-sectional FESEM images and (c) friction coefficient curve of CS3, which are deposited through the traditional method of ionizing argon gas and has the same experimental parameter with S3. [file 11671_2020_3354_MOESM1_ESM.docx]

**Supplementary material**


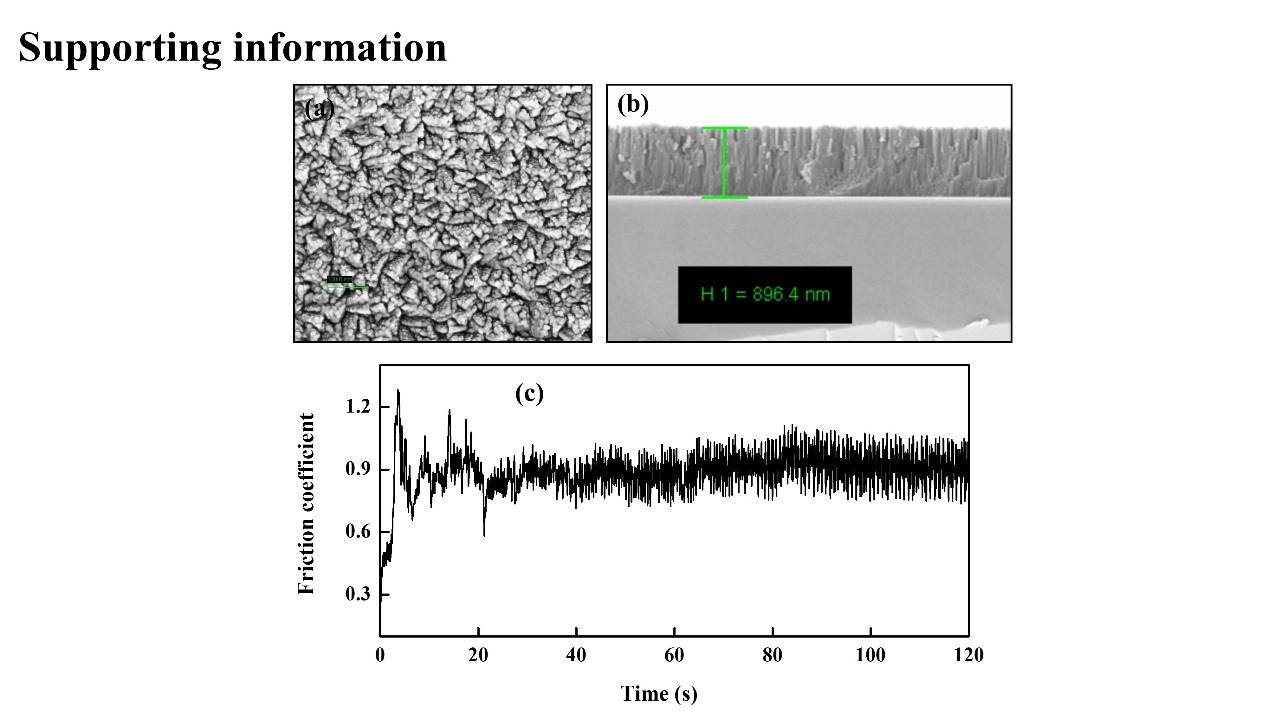


Fig.1 (a) Planar, (b)cross-sectional FESEM images and (c) friction coefficient curve of CS3, which are deposited through the traditional method of ionizing argon gas and has the same experimental parameter with S3.
